# Group I p21-activated kinases facilitate Tax-mediated transcriptional activation of the human T-cell leukemia virus type 1 long terminal repeats

**DOI:** 10.1186/1742-4690-10-47

**Published:** 2013-04-26

**Authors:** Ching-Ping Chan, Yeung-Tung Siu, Kin-Hang Kok, Yick-Pang Ching, Hei-Man Vincent Tang, Dong-Yan Jin

**Affiliations:** 1 Department of Biochemistry, The University of Hong Kong, 3/F Laboratory Block, 21 Sassoon Road, Pokfulam, Hong Kong; 2 Department of Anatomy and State Key Laboratory of Brain and Cognitive Science, The University of Hong Kong, 1/F Laboratory Block, 21 Sassoon Road, Pokfulam, Hong Kong

**Keywords:** HTLV-1, Tax, p21-activated kinases, CREB, CREB-regulating transcriptional activators

## Abstract

**Background:**

Human T-cell leukemia virus type 1 (HTLV-1) is the causative agent of adult T-cell leukemia and tropical spastic paraparesis. HTLV-1 encodes transactivator protein Tax that interacts with various cellular factors to modulate transcription and other biological functions. Additional cellular mediators of Tax-mediated transcriptional activation of HTLV-1 long terminal repeats (LTR) remain to be identified and characterized.

**Results:**

In this study, we investigated the regulatory role of group I p21-activated kinases (Paks) in Tax-induced LTR activation. Both wild-type and kinase-dead mutants of Pak3 were capable of potentiating the activity of Tax to activate LTR transcription. The effect of Paks on the LTR was attributed to the N-terminal regulatory domain and required the action of CREB, CREB-regulating transcriptional coactivators (CRTCs) and p300/CREB-binding protein. Paks physically associated with Tax and CRTCs. Paks were recruited to the LTR in the presence of Tax. siRNAs against either Pak1 or Pak3 prevented the interaction of Tax with CRTC1 and the recruitment of Tax to the LTR. These siRNAs also inhibited LTR-dependent transcription in HTLV-1-transformed MT4 cells and in cells transfected with an infectious clone of HTLV-1.

**Conclusion:**

Group I Paks augment Tax-mediated transcriptional activation of HTLV-1 LTR in a kinase-independent manner.

## Background

Human T-cell leukemia virus type 1 (HTLV-1) infects more than 20 million people worldwide and causes adult T-cell leukemia (ATL) or tropical spastic paraparesis (TSP) in a small subset of infected individuals. While ATL is a highly lethal malignancy of T lymphocytes, TSP is a disabling neuroinflammatory disorder of the central nervous system [1]. Although the development of ATL and TSP is rare and slow, high proviral load is the major risk factor for disease progression in carriers of HTLV-1 [2]. Understanding the mechanism by which HTLV-1 drives proviral expression might provide new strategies for prevention and control of HTLV-1-associated diseases.

The master regulator of HTLV-1 proviral expression is viral transactivator Tax which potently activates transcription from the long terminal repeats (LTR). For this activation, Tax forms a dimer to complex with dimeric CREB and the three imperfectly conserved Tax-responsive elements (TREs) in the LTR [3-5]. Tax also engages transcriptional coactivators such as p300/CREB-binding protein (CBP) and CREB-regulating transcriptional coactivators (CRTCs), also known as transducers of regulated CREB activity (TORCs) [5-10]. In addition, phosphorylation of Tax, CREB and CRTCs has also been implicated in LTR activation [10-14]. Because the activation of the LTR by Tax is a tightly regulated process accomplished through multiple mechanisms [4,5,15-17], we hypothesized that additional Tax-binding cellular proteins might also be involved in its execution and regulation. Particularly, it will be of great interest to see whether any Tax-binding protein kinases would play a role in this process.

p21-activated kinase 3 (Pak3) is one of the 32 Tax-binding proteins identified in a mass spectrometric analysis of proteins precipitated from HTLV-1-transformed C8166 cells using an anti-Tax antibody [18]. However, the roles of Pak3 in Tax-induced LTR activation have not yet been characterized. Paks are a family of serine-threonine kinases that regulate gene transcription, cell cycle progression, cytoskeletal organization and cell migration in response to small GTPases Cdc42 and Rac1 [19]. Pak signaling is commonly activated in cancer cells [20-22]. Paks are also implicated in the replication and spread of viruses such as human immunodeficiency virus type 1 (HIV-1) [23]. Currently there are six members in the Pak family that can be divided into group I (Pak1, Pak2 and Pak3) and group II (Pak4, Pak5 and Pak6) based on structural and biochemical properties. They have highly conserved Cdc42/Rac-interactive binding (CRIB) domain and kinase domain, but differ in tissue distribution and the N-terminal regulatory domain. Particularly, CRIB and kinase domains in Pak1, Pak2 and Pak3 share more than 95% identity [19]. It is noteworthy that Paks can fulfill some of their functions, such as the adaptor role in signal transduction, the inhibition of cell cycle progression as well as the induction of lamellipodia and membrane ruffling, in a kinase-independent manner [24-27].

In this study, we investigated the regulatory roles of group I Paks in Tax-induced activation of HTLV-1 LTR in transfected and infected cells. We found that all three Paks of group I can facilitate Tax-induced LTR activation. This activity of Paks was kinase-independent and was mediated through the N-terminal regulatory domain. Tax interacted with Paks and facilitated their recruitment to the viral promoter. Our work reveals new cellular mediators of HTLV-1 transcription and a new kinase-independent function of group I Paks in transcriptional regulation.

## Results

### Augmentation of Tax-induced activation of HTLV-1 LTR by group I Paks

Pak3 is known to interact with Tax in HTLV-1-transformed cells [18]. Within group I Paks, Pak1 and Pak2 are strikingly homologous to Pak3, sharing 80% and 69% identical amino acid residues [19,20]. While all three Paks in group I are activated by Cdc42/Rac to mediate similar biological effects, they are differentially expressed in different tissues and might have non-redundant functions by targeting different substrates. Particularly, Pak1 is abundantly expressed in brain, muscle and spleen. Pak2 is ubiquitous and Pak3 are primarily found in the brain [19]. Consistent with the previous finding on Pak3-Tax interaction in C8166 cells [18], we detected Pak1, Pak2 and Pak3 mRNA and protein in Jurkat cells and several lines of HTLV-1-transformed T cells, although the levels of Pak2 were relatively low (data not shown). With this in mind, we set out to explore whether Pak1, Pak2 and Pak3 might affect Tax-induced activation of HTLV-1 LTR.

**Figure 1:**
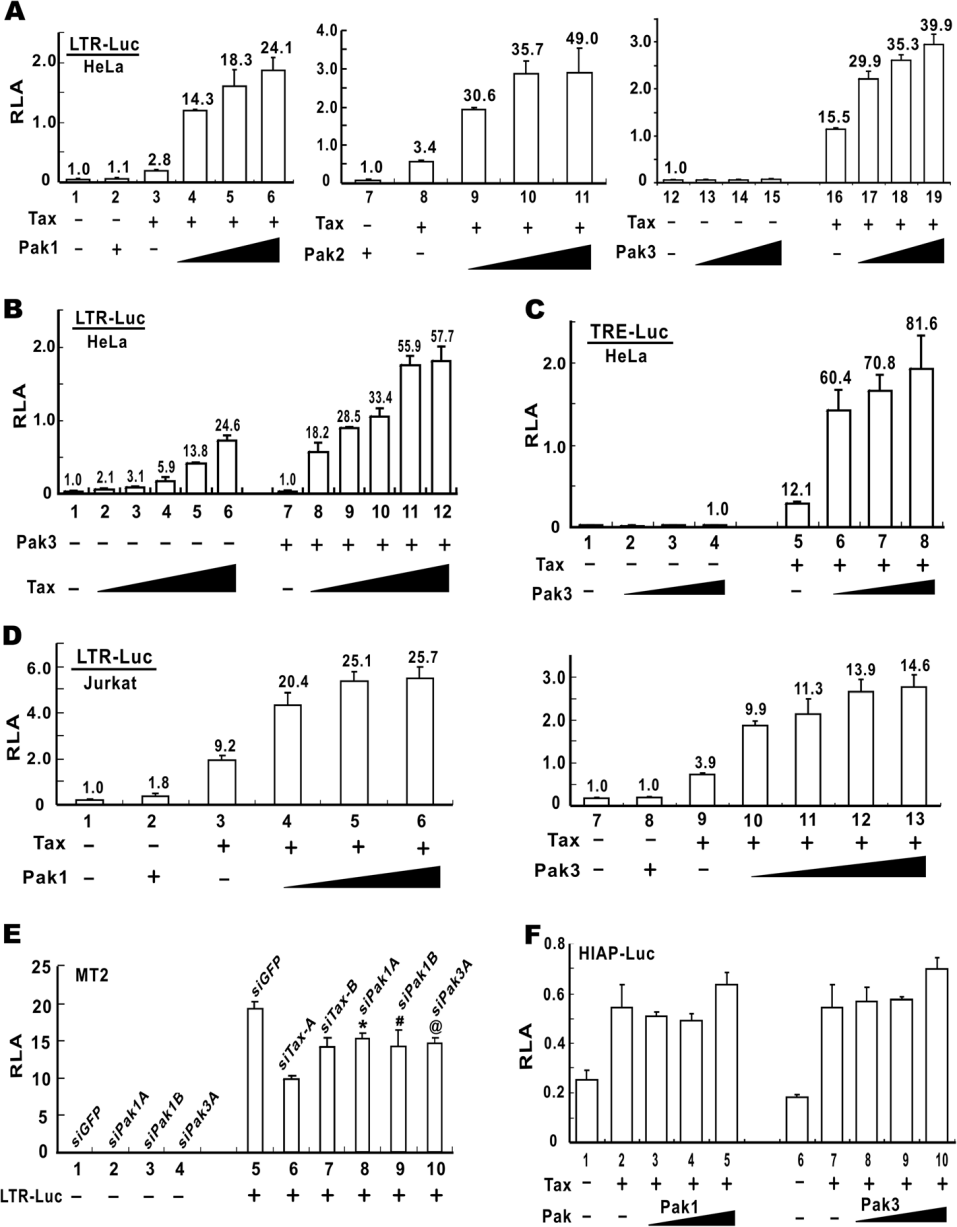
**Augmentation of Tax-induced activation of HTLV-1 LTR by group I Paks.** (**A** and **B**) Augmentation of LTR activation in HeLa cells. Cells were transfected with the indicated reporter and expression plasmids. A fixed amount of Tax expression plasmid plus escalating amounts of expression plasmids for Pak1/2/3 (A) or a fix amount of Pak3 expression plasmid plus escalating amounts of Tax plasmid (**B**) were used. Numbers at the top of the error bars indicate fold activation. (**C**) Augmentation of TRE activation in HeLa cells. The reporter plasmid pTRE-Luc contains three copies of TRE only. (**D**) Augmentation of LTR activation in Jurkat cells. (**E**) RNAi depletion of Pak1 or Pak3 dampened LTR activity in HTLV-1-transformed T cells. MT2 cells were transfected with pre-validated siRNA (100 nM) against Pak1 (siPak1A/B) or Pak3 (siPak3A). Cells were transfected with pLTR-Luc 30 h after siRNA transfection. Proliferation of MT2 cells were unaffected by siPak as measured by MTT assay. *: the difference between groups 6 and 5 is statistically significant (p = 0.0020 by Student's t test). #: p = 0.0017. @: p = 0.0018. (**F**) Paks have no influence on Tax-induced activation of NFκB in HeLa cells. Dual luciferase assays were performed. Relative luciferase activity (RLA) was calculated as a ratio of firefly luciferase activity recovered from pLTR-Luc or pHIAP-Luc versus that of *Renilla* luciferase recovered from co-transfected pSV-RLuc. Results represent three independent experiments and standard deviations (SD) are indicated by error bars.

We expressed Pak1, Pak2 and Pak3 in HeLa cells and assessed the influence on Tax-activated HTLV-1 LTR expression (Figure 1A). Although Pak1 did not affect basal activation of the LTR in the absence of Tax (Figure 1A, bar 2 compared to bar 1), a dose-dependent augmentation of Tax activity by Pak1 was observed (Figure 1A, bars 4–6 compared to bar 3). Likewise, Pak2 and Pak3 were also able to potentiate LTR activation by Tax (Figure 1A, bars 9–11 compared to bar 8, and bars 17–19 compared to bar 16). In contrast, neither Pak5 nor MEKK1 was capable of augmenting Tax-induced LTR activation (data not shown), suggesting that the effect is specific to group I Paks. This augmentation was more pronounced when Tax was limiting. For instance, Tax-induced LTR activation was increased 5–9 fold in the presence of Pak3 when Tax expression is low (Figure 1B, bars 8–10 compared to bars 2–4), whereas the enhancing effect of Pak3 was only 2–4 fold when Tax was abundant (Figure 1B, bars 11–12 compared to bars 5–6). Furthermore, Pak3 augmented Tax-induced activation of luciferase reporter expression driven by the 21-bp TREs alone (Figure 1C, bars 6–8 compared to bar 5). In support of this, the augmentation by Pak3 was not seen when the three TREs in the LTR were disrupted (data not shown). Thus, the TREs are both required and sufficient for the enhancing effect of Pak3 on Tax-induced activation of HTLV-1 LTR.

We next repeated the experiments in Jurkat cells and obtained similar results indicating the ability of Pak1 and Pak3 to further augment LTR activation by Tax (Figure 1D, bars 4–6 compared to bar 3, and bars 10–13 compared to bar 9). In addition, when we depleted endogenous Pak1 or Pak3 in Jurkat cells with a pre-validated siRNA, the activation of the LTR by Tax was compromised (Additional file 1: Figure S1A, bars 5 and 6 compared to bar 4). Consistent with this, Tax-mediated LTR activation was attenuated in HTLV-1-transformed MT2 and MT4 cells in which Pak1 or Pak3 was depleted by siRNA (Figure 1E, bars 8–10 compared to bar 5; Additional file 1: Figure S1B, bar 4 compared to bar 3). As a positive control, we also verified the suppression of LTR activation in Tax-depleted MT2 cells transfected with siTax-A or siTax-B (Figure 1E, bars 6 and 7 compared to bar 5). Since antibodies that can reproducibly detect endogenous Pak1 and Pak3 proteins in MT2 cells were not available, the silencing effect of siRNAs used was verified by RT-PCR analysis of Pak1 and Pak3 transcripts. Representative examples of RT-PCR results were presented in Additional file 2: Figure S2. Two independent siRNAs targeting Pak1 (siPak1A and siPak1B) were found to have a substantial suppressive effect on Pak1 mRNA expression in HeLa and MT4 cells (Additional file 2: Figure S2A, lanes 2 and 3 compared to lane 1; Additional file 2: Figure S2B, lanes 2 and 3 compared to lane 1). They also suppressed Pak3 mRNA expression to a lesser extent, but did not influence the expression of β-globin transcript (Additional file 2: Figure S2A, lanes 5 and 6 compared to lane 4; Additional file 2: Figure S2B, lane 5 compared to lane 4). Likewise, diminution of the steady-state levels of Pak3 mRNA was most pronounced in cells transfected with siPak3A and siPak3B (Additional file 2: Figure S2). The cross suppression of Pak1 and Pak3 by siPak3 and siPak1 respectively was due to the high homology between Pak1 and Pak3. Because the expression levels of Pak2 was low in MT2 and MT4 cells, RNAi depletion experiments were not performed for Pak2. Nevertheless, our results demonstrated that compromising group I Paks attenuated Tax-induced activation of HTLV-1 LTR.

In addition to HTLV-1 LTR, Tax can also activate NFκB-dependent transcription [28]. For example, Tax activates cellular HIAP promoter through NFκB [29]. Since Pak1 was also implicated in the activation of NFκB [30], we asked whether Pak1 or Pak3 might influence Tax-induced activation of NFκB. When we co-expressed Tax with Pak1 or Pak3, the activation of HIAP promoter by Tax was not further enhanced (Figure 1F, bars 3–5 compared bar 2, and bars 8–10 compared to bar 7). Additionally, when Pak1 or Pak3 was depleted from HeLa cells, Tax-induced activation of HIAP promoter was unaffected (data not shown). Hence, Pak1 and Pak3 had no influence on Tax-induced activation of NFκB. Collectively, our gain-of-function and loss-of-function experiments consistently supported the notion that group I Paks facilitate Tax-induced LTR activation specifically.

### Pak3 augments Tax-induced activation of HTLV-1 LTR in a kinase-independent manner

**Figure 2:**
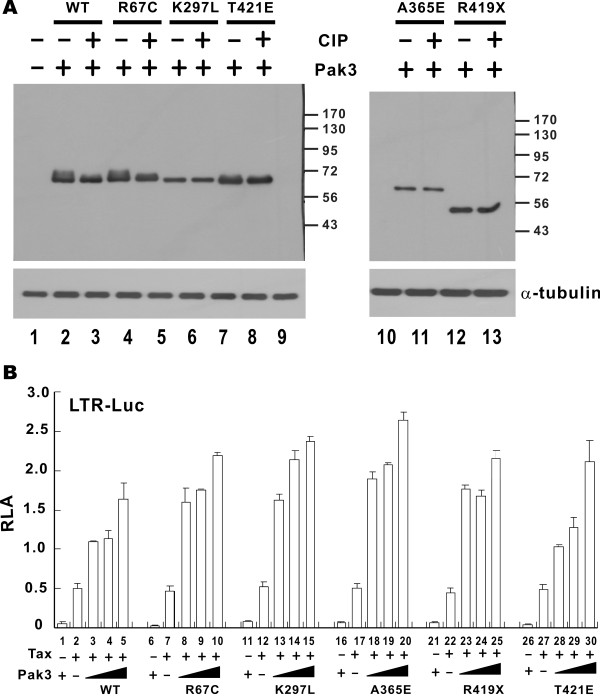
**Pak3 augments Tax-induced HTLV-1 LTR activation in a kinase-independent manner.** (**A**) Expression of wild-type and mutant forms of Pak3. HeLa cells were transfected with expression plasmids for the indicated Pak3 proteins. Cells were harvested 48 hrs post transfection. The harvested cells were untreated or treated with calf intestinal phosphatase (CIP) at 37°C for 30 min. Proteins were analyzed by Western blotting with anti-myc and anti-α-tubulin antibodies, respectively. (**B**) Activity profile of Pak3 wild-type and mutants on Tax-induced LTR activation. Dual luciferase assays were carried out as in Figure 1.

Augmentation of Tax-activated LTR activity by group I Paks raised the possibility for suppression of HTLV-1 transcription with small-molecule inhibitors of these kinases. As the first step to test this idea, we sought to determine the requirement of the kinase activity of Paks for the stimulatory effect on HTLV-1 LTR. Since Pak1, Pak2 and Pak3 are strikingly homologous, we focused our analysis on Pak3 only. We constructed various mutants of Pak3, some of which have been found in patients with X-linked mental retardation caused by a defect in Pak3 [31]. K297L, A365E and R419X mutants are kinase-defective, whereas T421E is a dominant active mutant. R67C is a mutant defective of Cdc42-binding [32]. All mutants were expressed to comparable levels (Figure 2A). Several slow-migrating bands were noted in the samples of Pak3 wild-type (WT) as well as R67C and T421E mutants (Figure 2A, lanes 2, 4 and 8), suggesting that they might be auto-phosphorylated. These slow-migrating bands disappeared when the samples were treated with calf intestinal phosphatase (Figure 2A, lanes 3, 5 and 9). For the kinase-dead K297L, A365E and R419X mutants, auto-phosphorylation of Pak3 was not observed (Figure 2A, lanes 6, 10 and 12). To our surprise, all mutants of Pak3 still augmented Tax-induced LTR activation equally well and in a dose-dependent manner (Figure 2B). Hence, kinase or Cdc42-binding activity of Pak3 was not essential for augmentation of Tax activity on the LTR.

**Figure 3:**
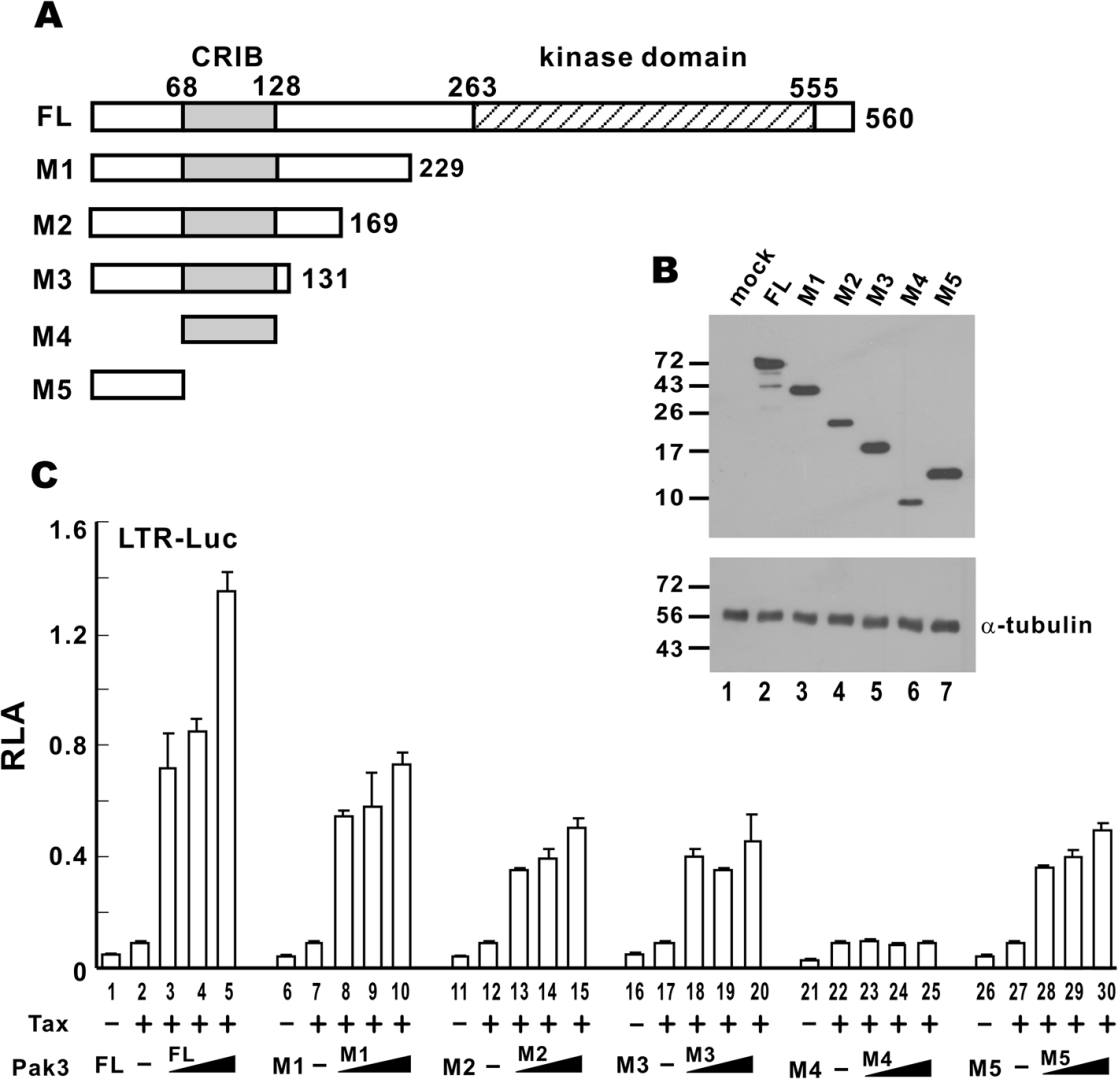
**Mapping the Tax-augmenting domain in Pak3.** (**A**) A diagram of Pak3 truncated mutants. (**B**) Expression of Pak3 mutants. Protein expression in HeLa cells was detected by Western blotting with anti-myc and anti-α-tubulin antibodies. (**C**) Activity profile of Pak3 truncated mutants on Tax-induced LTR activation. Dual luciferase assays were carried out as in Figure 1.

We went on to map the Tax-augmenting domain of Pak3 using a series of truncated mutants (Figure 3A). These mutants were expressed to comparable levels in HeLa cells (Figure 3B) and interrogated for the ability to potentiate Tax-induced activation of the LTR (Figure 3C). All mutants except M4, which contains the CRIB domain alone, were capable of augmenting LTR activation by Tax. These results indicated that the N-terminal regulatory domain (1–68 amino acids) was sufficient for augmentation of Tax-induced LTR activation. This is generally consistent with our findings obtained from the analysis of Pak3 point mutants (Figure 2).

To shed light on the functional domain of Tax that interacts with Pak3, we also examined the effect of Pak3 on various point mutants of Tax. Pak3 was found to augment all Tax mutants that are able to activate HTLV-1 LTR [33,34], including S258A, S150A, and C23S (Additional file 3: Figure S3). Thus, as in the case of many other Tax-binding proteins [35], we were unable to define a discrete domain of Tax that interacts with Pak3. Plausibly, the interaction is conformation-dependent and requires the full protein of Tax.

### CREB, CRTCs and p300/CBP are required for Tax-augmenting effect of Pak3

**Figure 4:**
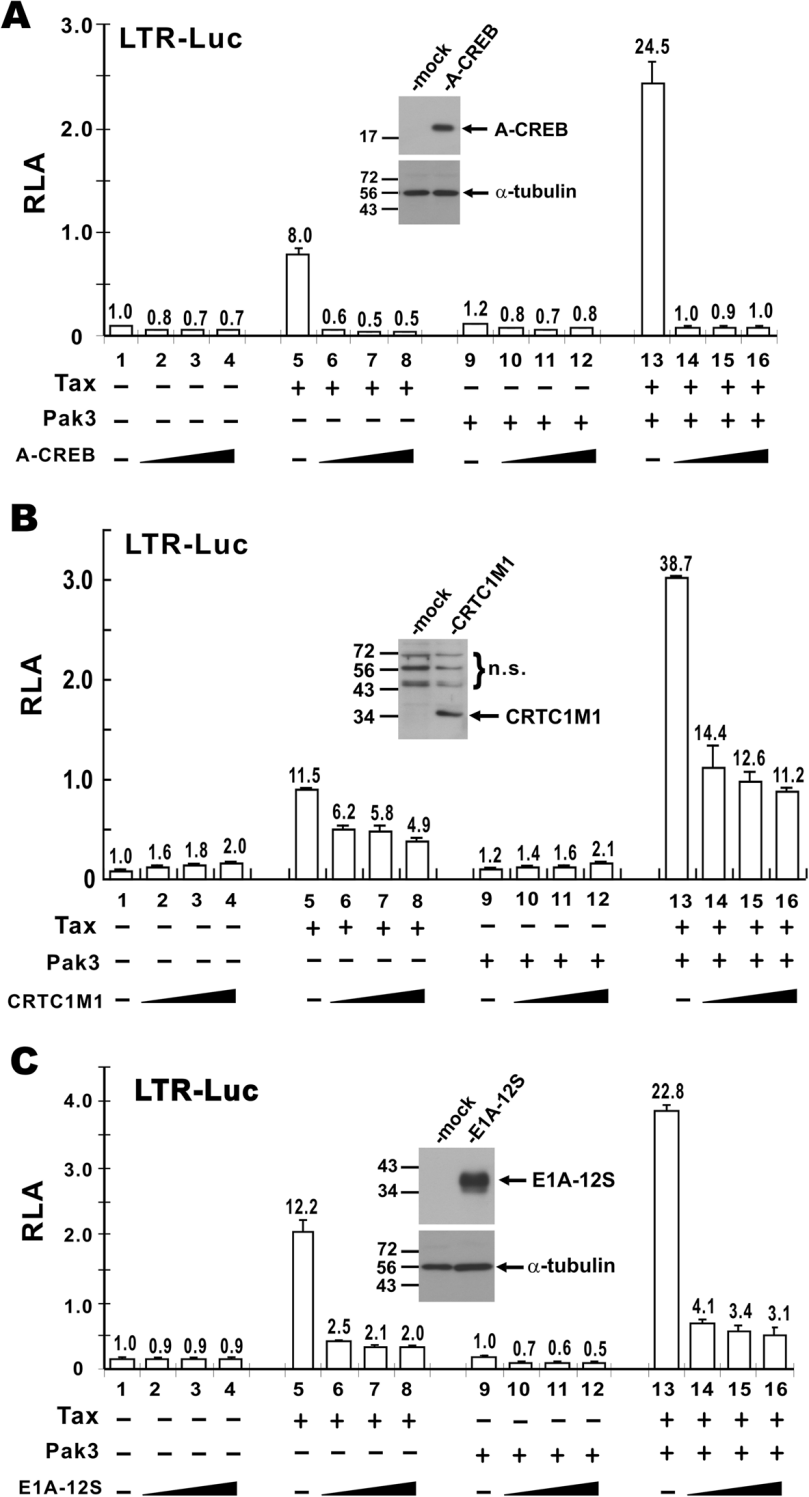
**Requirement of CREB, TORCs and p300/CBP for Pak3-augmented activation of HTLV-1 LTR by Tax.** Escalating amounts of A-CREB (**A**), CRTC1M1 (**B**), and E1A-12S (**C**) plasmids were transfected into HeLa cells. Dual luciferase assays were carried out as in Figure 1. Numbers at the top of the error bars indicate fold activation. Expression of A-CREB, CRTC1M1 and E1A-12S was verified by Western blotting with mouse anti-Flag, rabbit anti-Gal4 and rabbit anti-E1A antibodies (insets). n.s.: non-specific proteins reactive with anti-Gal4.

Tax-induced activation of HTLV-1 LTR is mediated through CREB transcription factor as well as CRTC and p300/CBP transcriptional coactivators [5,8]. Although Pak3 can augment Tax activity on the LTR, it is unclear whether the action of Pak3 would bypass any of these mediators. To address this issue, we employed dominant inactive proteins A-CREB and CRTC1M1 as well as an adenoviral inhibitor of p300/CBP known as E1A-12S. A-CREB and CRTC1M1 are well-characterized dominant inhibitory proteins that specifically impeded the function of CREB and CRTC1, respectively [4,36,37]. Adenoviral E1A-12S is capable of potently inhibiting the activity of p300, CBP and related transcriptional coactivators [38]. Expression of A-CREB, CRTC1M1 and E1A-12S even at a relatively low level is known to exert a dominant inhibitory effect on their specific targets [36-38]. Their expression in HeLa cells was verified by Western blotting (Figure 4 insets). Expression of A-CREB not only abrogated Tax-induced activation of the LTR, but also erased the Tax-augmenting effect of Pak3 (Figure 4A, bars 6–8 compared to bar 5, and bars 14–16 compared to bar 13). When similar sets of experiments were repeated with CRTC1M1, the activity of Tax on the LTR was also diminished both in the absence and in the presence of Pak3 (Figure 4B, bars 6–8 compared to bar 5, and bars 14–16 compared to bar 13), although the inhibition was incomplete plausibly due to redundant function of CRTC2 and CRTC3 [8]. Finally, inhibition of p300/CBP by E1A-12S effectively blunted the activity of Tax or Tax + Pak3 on the LTR (Figure 4C, bars 6–8 compared to bar 5, and bars 14–16 compared to bar 13). These results were compatible with the notion that CREB, CRTCs and p300/CBP are indispensable for Pak3-augmented activation of HTLV-1 LTR by Tax.

### Tax and CRTC1 associate with Paks

**Figure 5:**
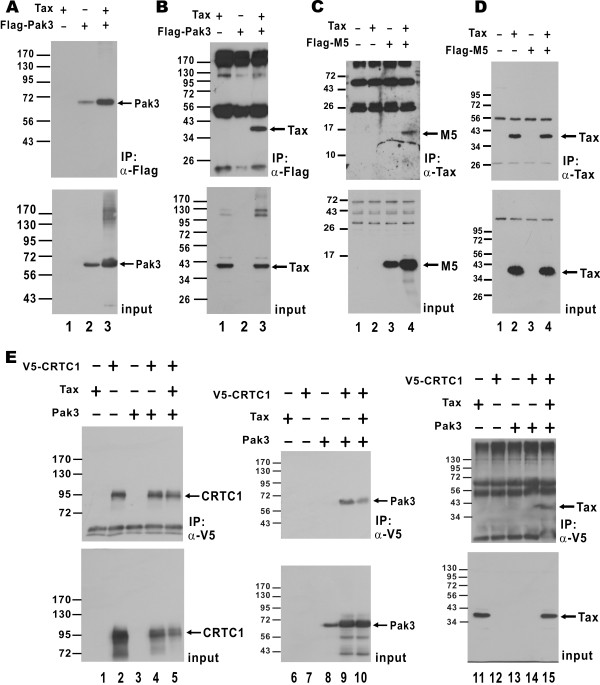
**Association of Pak3 with Tax and CRTC1 in cultured cells.** (**A** and **B**) Pak3 interacts with Tax. HEK293T cells were co-transfected with expression plasmids for Tax and Flag-Pak3. Pak3 was immunoprecipitated with anti-Flag. The precipitates were analyzed by Western Blotting using anti-Flag (**A**) and anti-Tax (**B**) to detect Pak3 and Tax, respectively. (**C** and **D**) M5 mutant of Pak3 associates with Tax. (**E**) Pak3 interacts with Tax and CRTC1. HEK293T cells were co-transfected with expression plasmids for Flag-Pak3, Tax and CRTC1-V5. CRTC1 was immunoprecipitated with anti-V5. CRTC1, Pak3 and Tax in the precipitates were detected with anti-V5, anti-Flag and anti-Tax antibodies, respectively.

Pak3 was previously shown to interact with Tax [18]. Since Pak1 shares 80% identical amino acid residues with Pak3 [19], we investigated whether Pak1 might also form a protein complex with Tax in cells. Using an anti-Flag (α-Flag) antibody, we precipitated Flag-tagged Pak1 from cultured HEK293T cells (Additional file 4: Figure S4A). In cells expressing both Tax and Pak1, Tax was found in the α-Flag precipitates (Additional file 4: Figure S4B, lane 3). However, the Tax-Pak1 complex was not detected in control cells expressing Tax or Pak1 alone (Additional file 4: Figure S4B, lanes 1 and 2). Likewise, the association between Pak3 and Tax was also confirmed (Figure 5A and B). Moreover, the M5 mutant of Pak3 containing the N-terminal regulatory domain alone (Figure 3A) was sufficient for association with Tax (Figure 5C and D). These results indicated that Pak1 and Pak3 can form a complex with Tax in cultured mammalian cells.

Tax interacts with Pak1 (Additional file 4: Figure S4B) and with CRTC1 individually [8]. In addition, Tax, Pak1 and CRTC1 can all be localized to the nucleus [8,39,40]. However, it was still unclear whether all three proteins might be found in the same protein complex. To investigate this, we expressed all three proteins in HEK293T cells and precipitated V5-tagged CRTC1 using an α-V5 antibody (Additional file 4: Figure S4C). The α-V5 precipitates contained both Pak1 and Tax (Additional file 4: Figure S4C, lanes 6 and 9). This was consistent with the formation of a protein complex that contains CRTC1, Pak1 and Tax. We also noted the association of Pak1 with CRTC1 in the absence of Tax (Additional file 4: Figure S4C, lane 5). Similar results compatible with the formation of CRTC1-Pak3 and CRTC1-Pak3-Tax protein complexes were also obtained (Figure 5E, lanes 9, 10 and 15). Hence, Pak1 and Pak3 associate with Tax and CRTC1.

### Paks are recruited to HTLV-1 LTR

**Figure 6:**
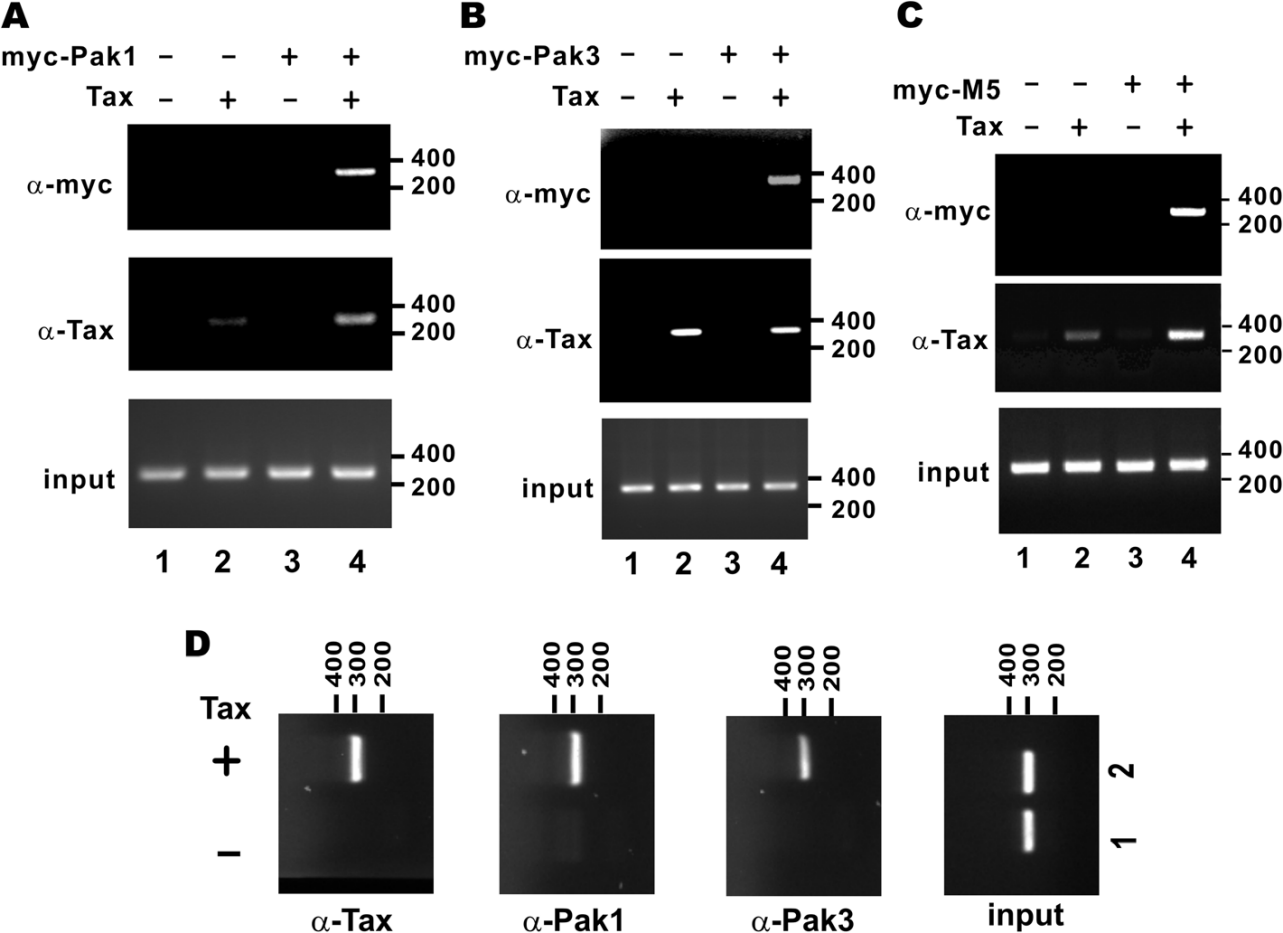
**Recruitment of Paks to HTLV-1 LTR in the presence of Tax.** (**A** to **C**) Recruitment of Pak1 (**A**), Pak3 (**B**) and Pak3-M5 (**C**) was assessed by ChIP. HeLa cells carrying LTR-Luc were co-transfected with expression vectors for Tax and myc-Pak1/3 or myc-M5 as indicated. After 48 h, the transfected cells were subjected to cross-linking with 1% formaldehyde for 10 min at 37°C. Cells were washed and then lysed with lysis buffer supplemented with protease inhibitor cocktail. The DNA-protein complex was sonicated, and the cell debris was removed by centrifugation. Myc-tagged Pak1/3-Tax-LTR promoter complex was immunoprecipitated using anti-myc or anti-Tax antibody and the LTR sequence was PCR-amplified. (**D**) Recruitment of endogenous Pak1 and Pak3. HeLa cells were co-transfected with Tax expression plasmid and pLTR-Luc. ChIP was performed with the indicated antibodies and the LTR sequence was PCR-amplified from the precipitated DNA-protein complex as above.

The association of Paks with Tax and CRTC1 plausibly in the nucleus raised the possibility that Paks might also be recruited to HTLV-1 LTR. To test this idea, we checked for the binding of Tax and Paks to HTLV-1 LTR using ChIP assay (Figure 6). We noted that overexpression of Pak1 or Pak3 alone in HeLa cells constitutively carrying HTLV-1 LTR was not sufficient to induce the recruitment of either kinase to the LTR (Figure 6,A and B, upper panels, lanes 3). However, when Pak1/3 and Tax were coexpressed, a more pronounced DNA band derived from the three 21-bp TREs in the LTR was amplified from the Pak1/3-DNA and Tax-DNA complexes (Figure 6,A and B, upper and middle panels, lanes 4). Likewise, the M5 mutant of Pak3 capable of interacting with and activating Tax (Figure 3C and Figure 5D) was also recruited to the LTR in the presence of Tax (Figure 6C, upper and middle panels, lanes 4). Furthermore, expression of Tax alone in HeLa cells resulted in the recruitment of endogenous Pak1 and Pak3 to the LTR (Figure 6D, lanes 2 compared to lanes 1). These results were compatible with the model that Tax facilitates the recruitment of Paks to HTLV-1 LTR.

### Paks are required for optimal Tax activity

**Figure 7:**
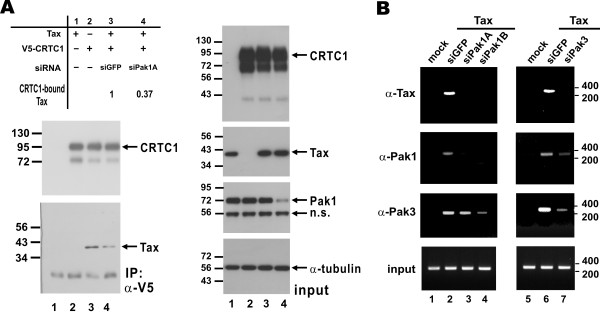
**Compromising Paks prevents CRTC1-Tax association and Tax recruitment to LTR.** (**A**) RNAi depletion of endogenous Pak1 impedes the association of Tax with CRTC1. HeLa cells were transfected with the indicated expression plasmids and an siRNA against Pak1 (siPak1A). Interaction of Tax with CRTC1 was analyzed as in Figure 5. n.s.: non-specific protein reactive with anti-Pak1. (**B**) RNAi depletion of Pak1 and Pak3 perturbs the recruitment of Tax to HTLV-1 LTR. HeLa cells were firstly transfected with siGFP, siPak1 and siPak3. siGFP served as a negative control. After 24 h of siRNA transfection, cells were transfected with Tax expression plasmid and pLTR-Luc. Cells were harvested for ChIP assay using the indicated antibodies after another 48 h.

To shed light on the requirement of Paks for the activity of Tax to interact with CRTCs and to be recruited to the LTR, we performed loss-of-function assays with siPak1/3 in HeLa cells. We first verified that the expression of endogenous Pak1, but neither CRTC1 nor Tax, was efficiently suppressed by siPak1A (Figure 7A, right panels, lanes 4 compared to lanes 3). We then pulled down CRTC1-containing complex with a monoclonal anti-V5 antibody and Tax was detected in this complex (Figure 7A, left panels). Notably, the relative amount of CRTC1-bound Tax was significantly reduced in Pak1-depleted cells (Figure 7A, left lower panel, lane 4 compared to lane 3). Thus, Pak1 is indispensible for optimal interaction of Tax with CRTC1.

We next assessed LTR recruitment of Tax in Pak1/3-depleted cells. As anticipated, suppression of Pak1/3 expression in HeLa cells by siPak1/3 also compromised their recruitment to the LTR (Figure 7B, middle panels). In addition, LTR recruitment of Tax was also suppressed in siPak1/3-transfected cells (Figure 7B, upper panel, lanes 3 and 4 compared to lane 2, and lane 7 compared to lane 6). These results suggest that group 1 Paks are also necessary for Tax recruitment to HTLV-1 LTR.

### Paks are required for optimal HTLV-1 transcription

**Figure 8:**
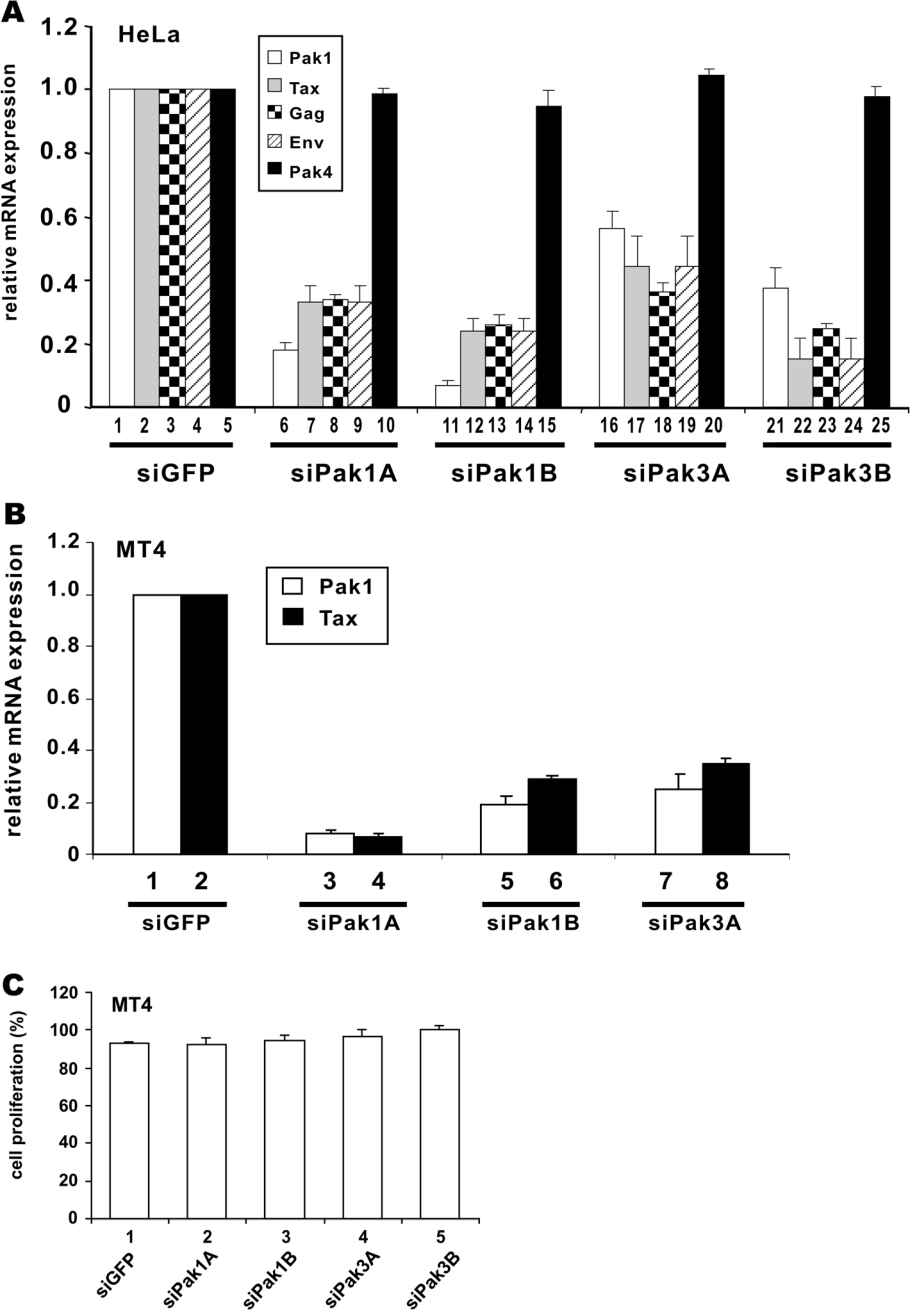
**Compromising Paks suppresses HTLV-1 proviral gene transcription.** (**A**) RNAi depletion of endogenous Pak1 and Pak3 decreases the expression of HTLV-1 proviral mRNA transcripts. HeLa cells were transfected with siPak1 or siPak3 to knockdown endogenous Pak1 and Pak3, respectively. After 24 h, cells were transfected with HTLV-1 molecular clone pX1MT. Cells were harvested 48 h after pX1MT transfection. Total RNA was extracted by Trizol and cDNA was synthesized. Quantitative RT-PCR was performed to analyze the relative levels of proviral Tax, Gag, Env, Pak1, Pak4 and β-globin transcripts. siGFP was used as a negative control. Relative expression levels were analyzed by normalizing to β-globin and fold difference represent 2^–ΔΔCT^. (**B**) RNAi depletion of Pak1 and Pak3 decreases the expression of Tax transcript in HTLV-1-transformed T cells. MT4 cells were transfected with siGFP, siPak1 and siPak3. siGFP was used as a negative control. After 72 h of siRNA transfection, total RNA was extracted and quantitative RT-PCR was performed. (**C**) Proliferation of MT4 cells was unaffected by siPak1/3 as measured by the MTT assay.

Above we have characterized the ability of group I Paks to augment Tax-induced LTR activation. We have also demonstrated the requirement of Paks for optimal activity of Tax to interact with CRTC1 and to be recruited to the LTR. If Paks indeed function to facilitate Tax in HTLV-1-infected cells, compromising Paks should exert a suppressive effect on HTLV-1 proviral transcription. With this in mind, we depleted Pak expression in HeLa cells with siRNA and transfected subsequently with pX1MT, an infectious clone of HTLV-1 that can drive the expression of viral proteins and the production of infectious virions [41]. The expression of viral and cellular transcripts was then monitored by real-time RT-PCR. siPak1 and siPak3 were effective in dampening the expression of Pak1, but not Pak4 (Figure 8A, bars 6, 11, 16 and 21 compared to bar 1, and bars 10, 15, 20 and 25 compared to bar 5). All four siPaks were also found to substantially inhibit the expression of Tax, Gag and Env transcripts (Figure 8A, bars 7–9, 12–14, 17–19 and 22–24 compared to bars 2–4). Thus, Paks are required for optimal transcriptional activation of HTLV-1 by Tax.

On the other hand, we also measured the expression of Pak1 and Tax transcripts in HTLV-1-transformed and Tax-expressing MT4 cells transfected with siPaks (Figure 8B). Again, silencing of Pak1 or Pak3 correlated with down-regulation of Tax expression (Figure 8B, bars 3–8), but did not apparently affect cell proliferation (Figure 8C). Taken together, these results provided further support to the notion that group I Paks are facilitators of Tax-activated HTLV-1 transcription in both acutely and chronically infected cells.

## Discussion

In this study, we provided the first evidence for a facilitator function of group I Paks in Tax-induced activation of HTLV-1 LTR. This kinase-independent function of Pak1, Pak2 and Pak3 was characterized in the context of Tax and HTLV-1 LTR (Figures 1 and 2). CREB, CRTCs, p300/CBP and the N-terminal regulatory domain of Paks are indispensable for this function (Figures 3, 4, 5 and 6). Paks associate with Tax and CRTCs and are recruited to HTLV-1 LTR by Tax (Figures 5, 6 and Additional file 4: Figure S4). Compromising Paks by RNAi resulted in a suppression of the interaction of Tax with CRTC1 and the recruitment of Tax to the LTR (Figure 7), plausibly leading to an inhibition of HTLV-1 transcription in cells infected with an HTLV-1 clone and in HTLV-1-transformed T cells (Figures 1, 8, Additional file 1: Figures S1 and Additional file 2: Figures S2). Collectively, our findings reveal new mechanistic details of Tax-induced transcriptional activation of HTLV-1 LTR, in which Tax interacts with and recruits group I Paks to the TRE enhancers to facilitate transcription.

Group I Paks are Cdc42/Rac-regulated kinases that regulate transcription, cell cycle, cell motility and other aspects of cell physiology [19,20]. Some functions of Paks do not require their kinase activity [24-27]. For example, Pak1 induces lamellipodia formation and membrane ruffling through its N-terminal regulatory domain in a kinase-independent fashion [24,25]. Inhibition of cell cycle progression by the CRIB domain of Pak1 is also independent of its kinase activity [26]. In addition, Pak1 serves a kinase-independent scaffolding role in Akt signaling [27]. However, it is still surprising that group I Paks augment Tax transcriptional activity on HTLV-1 LTR in a kinase-independent manner. Exactly how these Paks facilitate Tax in LTR activation remains to be elucidated. The diminution of CRTC1- and LTR-bound Tax in Pak1/3-compromised cells (Figure 7) was in support of an adaptor function of group I Paks in Tax-induced activation of the LTR. On the other hand, Pak1 was previously shown to stimulate transcription when tethered to the promoter through Gal4 DNA-binding domain (Gal4BD) [37]. One alternative mechanism by which Pak1 modulates transcription is through inactivation of transcriptional corepressor CtBP [42], which inhibits the function of CBP [43]. Pak1 also interacts with and phosphorylates histone H3 [44]. We found that the M5 mutant of Pak3 containing the N-terminal regulatory domain alone could sufficiently interact with Tax, promote Tax recruitment to the LTR, and augment Tax-induced LTR activation (Figures 3, 5 and 6). Although neither CRIB nor kinase activity was required for the augmentation of Tax activity (Figures 2 and 3), we cannot completely rule out the involvement of the kinase domain since the Tax-augmenting activity of M1 lacking the kinase domain was weakened compared to the wild type (Figure 3). Our experiments suggested that the augmentation of Tax activity by group I Paks could not bypass CREB, CRTCs or p300/CBP (Figure 4). However, our analysis could not distinguish the effect of the dominant inhibitors of CREB, CRTC1 and p300/CBP on Tax or on Paks. Particularly, the incomplete inhibitory effect of CRTC1M1 (Figure 4B) suggested that compromising CRTC1 alone is insufficient to abrogate the activity of Tax and Pak3. It will be of interest to determine whether CRTC2 and CRTC3 could account for the remaining activity of Tax and Pak3 in the presence of CRTC1M1. Exactly how Paks affect the activity of CREB, CRTCs and p300/CBP remains to be elucidated. In this regard, we showed that depletion of Pak1 suppressed the recruitment of Tax to the LTR (Figure 7B). Because Tax is required for the recruitment and activation of CREB, CRTCs and p300/CBP [5-9], the activity of these transcriptional regulators should also be compromised in the absence of Paks. At least three additional lines of experiments are required to derive mechanistic insight on the transcriptional regulatory function of group I Paks. First, the dispensability of the kinase domain for the augmentation of Tax activity should be determined. Second, the M5 mutant recruited to the promoter through Gal4BD should be assessed for transcriptional activity. Finally, possible involvement of CtBP and histone H3 phosphorylation in Pak-augmented activation of HTLV-1 LTR by Tax should be investigated.

Emerging evidence implicates group I Paks in supporting the replication of various human viruses [23]. Thus, association and activation of Pak2 by viral Nef protein were thought to play a role in HIV-1 replication [45]. In addition, an RNAi screen identified Pak1 and Pak3 to be important host factors that support HIV-1 infection in HeLa cells and T lymphocytes [46]. On the other hand, hepatitis B virus oncoprotein HBx was recently shown to activate Pak1 to promote cell survival [47]. Our findings that group I Paks facilitate HTLV-1 transcription are generally consistent with the notion that these Paks play an important role in the life cycle of human viruses of different families. Small-molecule inhibitors of Paks are thought to be attractive therapeutic agents for different types of cancer and viruses [48]. Because the facilitator function of Paks on Tax-induced LTR activation is kinase-independent, kinase inhibitors of Paks might not be useful in anti-HTLV-1 therapy. However, peptide mimetics that can inhibit the interaction between Paks and Tax would still hold the promise for the design and development of new molecularly targeted anti-HTLV-1 and anti-ATL therapeutics.

Group I Paks are a critical regulatory point in cell signaling on which diverse upstream signals converge [18,19]. It remains to be understood how these signals might affect the interaction between Tax and Paks. Tax appears to be able to promote the recruitment of Paks to HTLV-I LTR (Figure 6). The mechanisms of this recruitment and the cellular factors that regulate this process warrant further investigations. Paks are frequently upregulated in cancer cells and virus-infected cells [19,23]. In line with this, Pak1 and Pak3 were found to be expressed in HTLV-1-transformed leukemic cells and compromising their expression led to inhibition of HTLV-1 transcription (Figures 1, 8, Additional file 1: Figure S1 and Additional file 2: Figure S2). To understand whether group I Paks might indeed be induced by HTLV-1 infection, expression profiles of Pak mRNA and protein in HTLV-1-infected individuals and ATL patients should be determined systematically. Pak1, Pak2 and Pak3 are strikingly homologous and serve redundant and non-redundant functions in cells [18,19]. We found that all three were equally active in facilitating Tax-induced LTR activation (Figure 1). Comparing their relative abundance in HTLV-1-infected T cells in patients will shed light on whether they are differentially expressed and which ones might be more important in HTLV-1 transcription.

The activation of CREB signaling by Tax not only mediates LTR activation, but is also required for full-blown oncogenic transformation [15,49]. In addition to the LTR, Tax also activates a wide array of cellular genes through CREB to effect cell proliferation and survival [47,48]. We noted in our study that Pak1 and Pak3 associated with CRTC1 even in the absence of Tax (Figures 5 and Additional file 4: Figure S4), raising the possibility that group I Paks might serve a general facilitator function in cellular CREB-dependent transcription. That is to say, group I Paks could act as cofactors in CREB-induced cellular transformation. In HTLV-1-infected cells, Tax might further enhance the activity of Paks to facilitate CREB-dependent transcription by recruiting them to CRE-containing promoters. In this regard, full characterization of the role of group I Paks in the activation of cellular CREB-regulated genes both in the absence and in the presence of Tax will enable us to have a complete picture of how Tax, group I Paks and CREB cooperate to mediate transcriptional activation and oncogenic transformation.

## Conclusion

We demonstrate that group I Paks interact with HTLV-1 Tax and are recruited to the LTR to serve a kinase-independent facilitator function in Tax-induced activation of LTR transcription.

## Methods

### Cell culture and transfection

HeLa and HEK293T cells were cultured in Dulbecco's modified Eagle's medium supplemented with 10% fetal bovine serum. Jurkat and HTLV-1-transformed T cells MT2 and MT4 were maintained in RPMI medium supplemented with 10% fetal bovine serum. HeLa and HEK293T cells were transfected using GeneJuice transfection reagent (Novagen). Jurkat, MT2 and MT4 cells were transfected using Lipofectamine 2000 (Invitrogen).

### RNA interference

RNA knockdown experiments were carried out as described [51,52]. HeLa, Jurkat, MT2 and MT4 cells were transfected with 100 nM siRNA using Lipofectamine 2000 (Invitrogen). siRNA sequences are as follows:

siPak1A: 5′-GCUCUGUCAA GCUAACUGAdT dT-3′

siPak1B: 5′-GCUCUGGAGU UCUUGCAUUdT dT-3′

siPak3A: 5′-CAACCCAAGA AGGAAUUAAdT dT-3′

siPak3B: 5′-CCAGAUCACU CCUGAGCAAdT dT-3′

siTax-A: 5′-GGCCUCAUAC AGUACUCUUdT dT-3′

siTax-B: 5′-GGCAGAUGAC AAUGACCAUdT dT-3′

siGFP: 5′-GAAGCAGCAC GACUUCUUCdT dT-3′

### Plasmids

Reporter plasmid pLTR-Luc and expression plasmids for Tax, A-CREB, CRTC1, CRTC1M1 and Pak1 were described elsewhere [4,8,21,36,37]. pLTR-Luc contains the full LTR of HTLV-1. Reporter plasmid pTRE-Luc was constructed by inserting into pGL3-basic (Promega) three copies of TREs amplified from HTLV-1 LTR using primers 5′-CCCAAGCTTG GTCAGGGCCC AGACTAAGGC TC-3′ and 5′-CCCAAGCTTT GGATGGCGGC CTCAGGTAAG G-3′. Reporter plasmid pHIAP-Luc was a gift from R. Grassmann [29]. Expression plasmid for E1A-12S was provided by J. Lundblad [38]. HTLV-1 molecular clone pX1MT was provided by D. Derse [41]. Pak2 expression construct was provided by W. Hahn [53] and Pak2 was subcloned using primers 5′-GGGGTACCAT CATGTCTGAT AACGGAGAAC TGGAAG-3′ and 5′-ATAAGAATGC GGCCGCTTAA CGGTTACTCT TCATTGCTTC TTT-3′. Pak3 was derived from I.M.A.G.E. cDNA clone 4798769 and was amplified using primers 5′-GCGGATCCAG ATGTCTGACG GTCTGGATA ATG-3′ and 5′-CCGCTCGAGT TAGCGGCTGC TGTTCTTAAT TGCTTCC-3′. R67C, K297L, A365E, R419X and T421E mutants of Pak3 were constructed by PCR method using the following sets of sense (s) and antisense (as) primers (the mutated nucleotides are underlined):

R67C-s: 5′-CCAATAAGAA GAAAGAGAAA GAGTGCCCAG AGATCTCTCT TCC-3′

R67C-as: 5′-GGAAGAGAGA TCTCTGGGCA CTCTTTCTCT TTCTTCTTAT TGG-3′

K297L-s: 5′-GAGGTGGCCA TACTGCAGAT GAACCTTCAA CAGC-3′

K297L-as: 5′-GCTGTTGAAG GTTCATCTGC AGTATGGCCACC TC-3′

A365E-s:  5′-CCTGTATGGA TGAAGGACAG ATAGAAGCTG TCTG-3′

A365E-as: 5′-CAGACAGCTT CTATCTGTCC TTCATCCATA CAGG-3′

R419X-s: 5′-CTGAGCAAAG TAAATGAAGC ACTATGGTGG GAAC-3′

R419X-as: 5′-GTTCCCACCA TAGTGCTTCA TTTACTTTGC TCAG-3′

T421E-s: 5′-CTGAGCAAAG TAAACGAAGC GAGATGGTGG GAAC-3′

T421E-as: 5′-GTTCCCACCA TCTCGCTTCG TTTACTTTGC TCAG-3′

Truncated mutant M4 of Pak3 was made by PCR using primers 5′-GCGGATCCAG ATGCGCCCAG AGATCTCTCT TCCTTCA-3′ and 5′-CCGCTCGAGT TACTTCTTCT GTTCCAATTT TGTTATGTTG G-3′. Truncated mutants M1, M2, M3 and M5 of Pak3 were generated by PCR using the common sense primer 5′-GCGGATCCAG ATGTCTGACG GTCTGGATAA TG-3′ and the following anti-sense primers:

M1-as: 5′-CCGCTCGAGT TATGCTGGTG AAGCAATGGA TTCAACCAC-3′

M2-as: 5′-CCGCTCGAGT TAATGGGCTG CTATGTATCC ATGTGCACT-3′

M3-as: 5′-CCGCTCGAGT TATGGGTTCT TCTTCTGTTC CAATTTTGTT AT-3′

M5-as: 5′-CCGCTCGAGT TAGATCTCTG GGCGCTCTTT CT-3′

### Luciferase reporter assays

Dual luciferase assay was carried out as previously described [8,37]. Transfection efficiencies were normalized to pSVRL control plasmid expressing *Renilla* luciferase (Promega).

### RT-PCR analysis

RNA was extracted using Trizol reagent (Invitrogen). RNA (2 μg) was digested with DNase I (Ambion) at 37°C for 30 min. cDNA synthesis was performed using oligo (dT). Semi-quantitative RT-PCR was performed as previously described [37,51]. Primer sets were as follows:

Tax-s: 5′-TCTCACACGG CCTCATACAG-3′

Tax-as: 5′-ATATTTGGGG CTCATGGTCA-3′

Gag-s: 5′-CTTTGCTCCT CCCTCGTG-3′

Gag-a: 5′-TTGCTGGTAT TCTCGCCTTA-3′

Env-s: 5′-TGGCGGAGGC TATTATTCAG-3′

Env-as: 5′-TTGAGGCGTGACACTTCTTG-3′

XII-s: 5′-CGGATACCCA GTCTACGTGT TTG-3′

XII-as: 5′-GGGAGTCGAG GGATAAGGAA CT-3′

Pak1-s: 5′-TGAGAGCCTT GTACCTCATT GCCA-3′

Pak1-as: 5′-TCCTTAGCTG CAGCAATCAG TGGA-3′

Pak3-s: 5′-ACAACCGGGA TTCTTCAGCA CTCA-3′

Pak3-as: 5′- AGTAATCGTG CCCATTGCTC TGGA-3′

globin-s: 5′-AGCGTACTCC AAAGATTCAG GTT-3′

globin-as: 5′-TACATGTCTC GATCCCACTT AACTAT-3′

Real-time RT-PCR was performed as previously described [37,50]. Relative expression levels were quantified by normalizing to the corresponding β-globin values using the comparative threshold cycle method where:

Fold difference = 2^–(ΔCT^^of gene of interest -^^ΔCT^^of^ β^-globin)^ = 2^–ΔΔCT^

Primer sets for quantitative RT-PCR were as follows:

Tax-s: 5′-TACTACAGTC CTCCTCCT-3′

Tax-as: 5′-CCCTCATTTC TACTCTCAC-3′

Pak1-s: 5′-GACATCCAAC AGCCAGAA-3′

Pak1-as: 5′-ACACAGCCTT CACATTCAA-3′

Gag-s: 5′-CTTTGCTCCT CCCTCGTG-3′

Gag-as: 5′-TTGCTGGTAT TCTCGCCTTA-3′

Env-s: 5′-TGGCGGAGGC TATTATTCAG-3′

Env-as: 5′-TTGAGGCGTG ACACTTCTTG-3′

Pak4-s: 5′-GGATAATGGTGATTGAGAT-3′

Pak4-as: 5′-ATCATCTTCATGGCTTTG-3′

### Co-immunoprecipitation

Co-immunoprecipitation was carried out as described [52,54]. HEK293T cells were lysed with lysis buffer (20 mM Tris–HCl, pH 7.5, 100 mM NaCl, 0.1% NP-40, and 0.5 mM EDTA) supplemented with protease inhibitors (Roche). Cell debris was removed by centrifugation at 14,000 rpm at 4°C. Cell lysate was incubated with primary antibodies at 4°C overnight. The immunocomplex was incubated with 30 μl protein A-agarose (Invitrogen), washed three times with lysis buffer, and then resuspended with SDS-PAGE loading buffer (60 mM Tris-Cl, 2% SDS, 6% glycerol, 1% β-mercaptoethanol, and 0.002% bromophenol blue).

### Chromatin immunoprecipitation

Chromatin immunoprecipitation (ChIP) was performed as previously described [37,55]. HeLa cells were cross-linked by 1% formaldehyde for 10 min at room temperature. The DNA–protein complex was immunoprecipitated, and the genomic DNA was purified by phenol-chloroform extraction. Promoter sequence spanning the three 21-bp TREs in HTLV-1 LTR was PCR-amplified using primers 5′-GGCTTAGAGC CTCCCAGTG-3′ and 5′-CTCCTGAACT GTCTCCACGC-3′.

### Cell proliferation assay

Cell proliferation assay was performed using the (3-(4,5-dimethylthiazol-2-yl)-2,5-diphenyl-tetrazolium (MTT) method [56]. MT4 cells (5 × 10^4^) were treated with 5 mg/ml MTT solution (10 μl) and OD_550_ was measured with a microplate reader (Spectra Max 340, Molecular Devices). Cell proliferation was presented as a percentage of the control.

## Competing interests

The authors declare that they have no competing interests.

## Authors’ contributions

CPC, YPC, HMVT and DYJ designed the experiments, analyzed data and wrote the manuscript; CPC, YTS and HMVT performed the experiments; and KHK analyzed data and provided advice. All authors read and approved the final manuscript.

## Supplementary Material

Additional file 1: Figure S1 Knockdown of Paks alleviates LTR activity in T cells. (A) Silencing of Paks represses LTR activation by Tax in Jurkat cells. Jurkat cells were transfected with siRNA (siPak1 or siPak3) to deplete endogenous Pak1 or Pak3. After 30 h, cells were co-transfected with expression vector for Tax and pLTR-Luc reporter plasmid. Dual luciferase activity was assayed as in Figure 1.*: the difference between groups 5 and 4 is statistically significant (p = 0.0013 by Student's t test). #: p = 0.0006. (B) Silencing of Pak1 alleviates LTR activity in MT4 cells. Cells were transfected with the indicated plasmids, and harvested for dual luciferase assay after 48 h. @: the difference between groups 4 and 3 is statistically significant (p = 0.013 by Student's t test). Click here for file

Additional file 2: Figure S2 Repression of HTLV-1 proviral gene transcription by depleting Pak1/3. HeLa (A) and MT4 (B) cells were co-transfected as in Figure 7. Semi-quantitative RT-PCR was performed. Indicated at the bottom of the panels are relative amounts of Pak1 or Pak3 transcript normalized to β-globin (β-glb) mRNA (*/@ and #/@) as determined by densitometric analysis of band intensity. Click here for file

Additional file 3: Figure S3 Pak3 augments LTR activation by Tax mutants. HeLa cells were transfected with the indicated plasmids and analyzed as in Figure 1. Click here for file

Additional file 4: Figure S4 Pak1 interacts with Tax and CRTC1 in HEK293T cells. Cells were co-transfected with expression vectors for Pak1, CRTC1 and Tax as indicated. Co-immunoprecipitation and Western blotting were performed as in Figure 5. Click here for file
