# Supplementary figures and images for: Group I p21-activated kinases facilitate Tax-mediated transcriptional activation of the human T-cell leukemia virus type 1 long terminal repeats
Source: Retrovirology. 2013 Apr 26;10:47. doi: 10.1186/1742-4690-10-47 (PMC3651266; doi:10.1186/1742-4690-10-47)

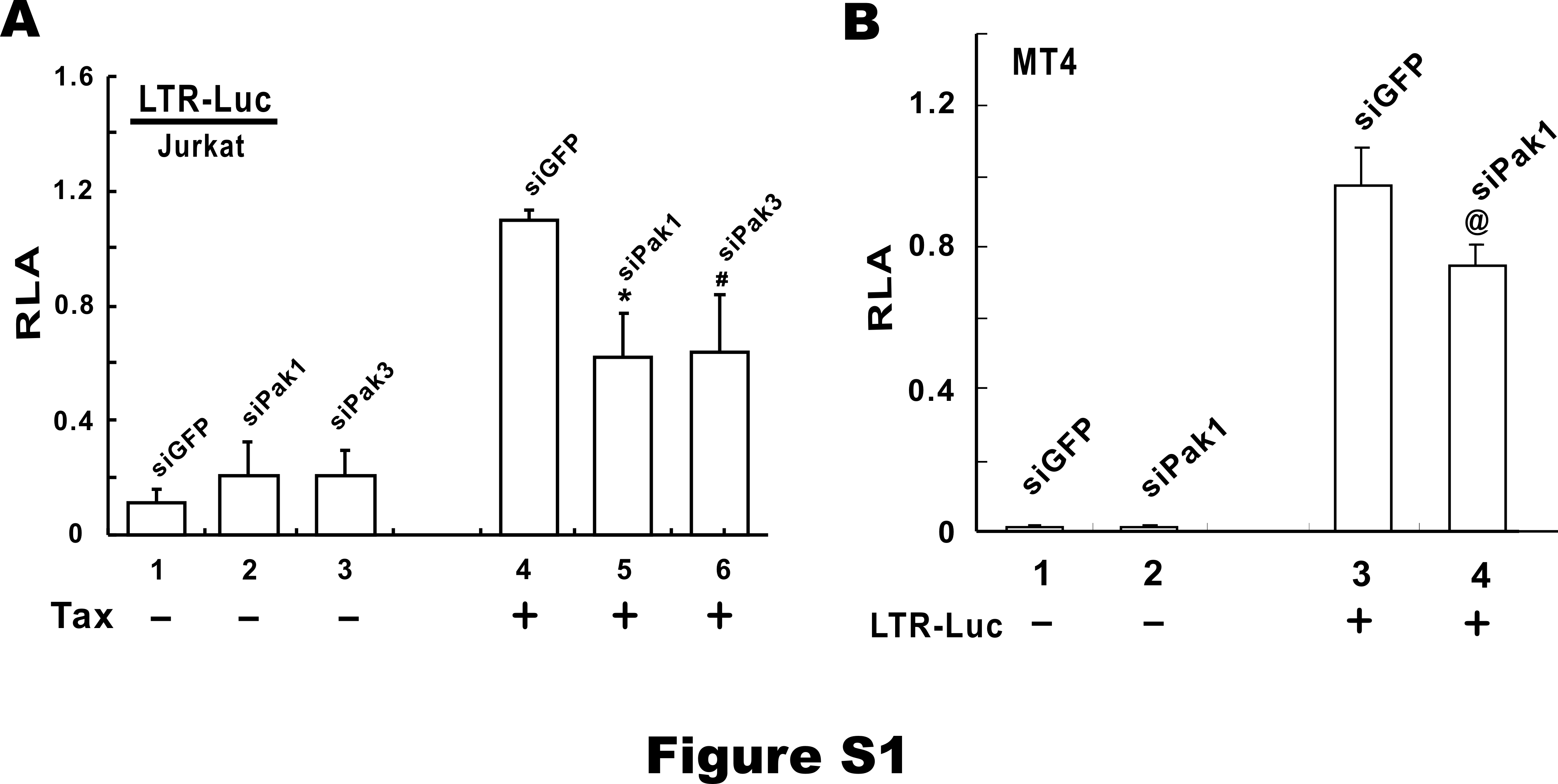

Supplement: Additional file 1: Figure S1 — Knockdown of Paks alleviates LTR activity in T cells. (A) Silencing of Paks represses LTR activation by Tax in Jurkat cells. Jurkat cells were transfected with siRNA (siPak1 or siPak3) to deplete endogenous Pak1 or Pak3. After 30 h, cells were co-transfected with expression vector for Tax and pLTR-Luc reporter plasmid. Dual luciferase activity was assayed as in Figure 1.*: the difference between groups 5 and 4 is statistically significant (p = 0.0013 by Student's t test). #: p = 0.0006. (B) Silencing of Pak1 alleviates LTR activity in MT4 cells. Cells were transfected with the indicated plasmids, and harvested for dual luciferase assay after 48 h. @: the difference between groups 4 and 3 is statistically significant (p = 0.013 by Student's t test). [file 1742-4690-10-47-S1.tiff]

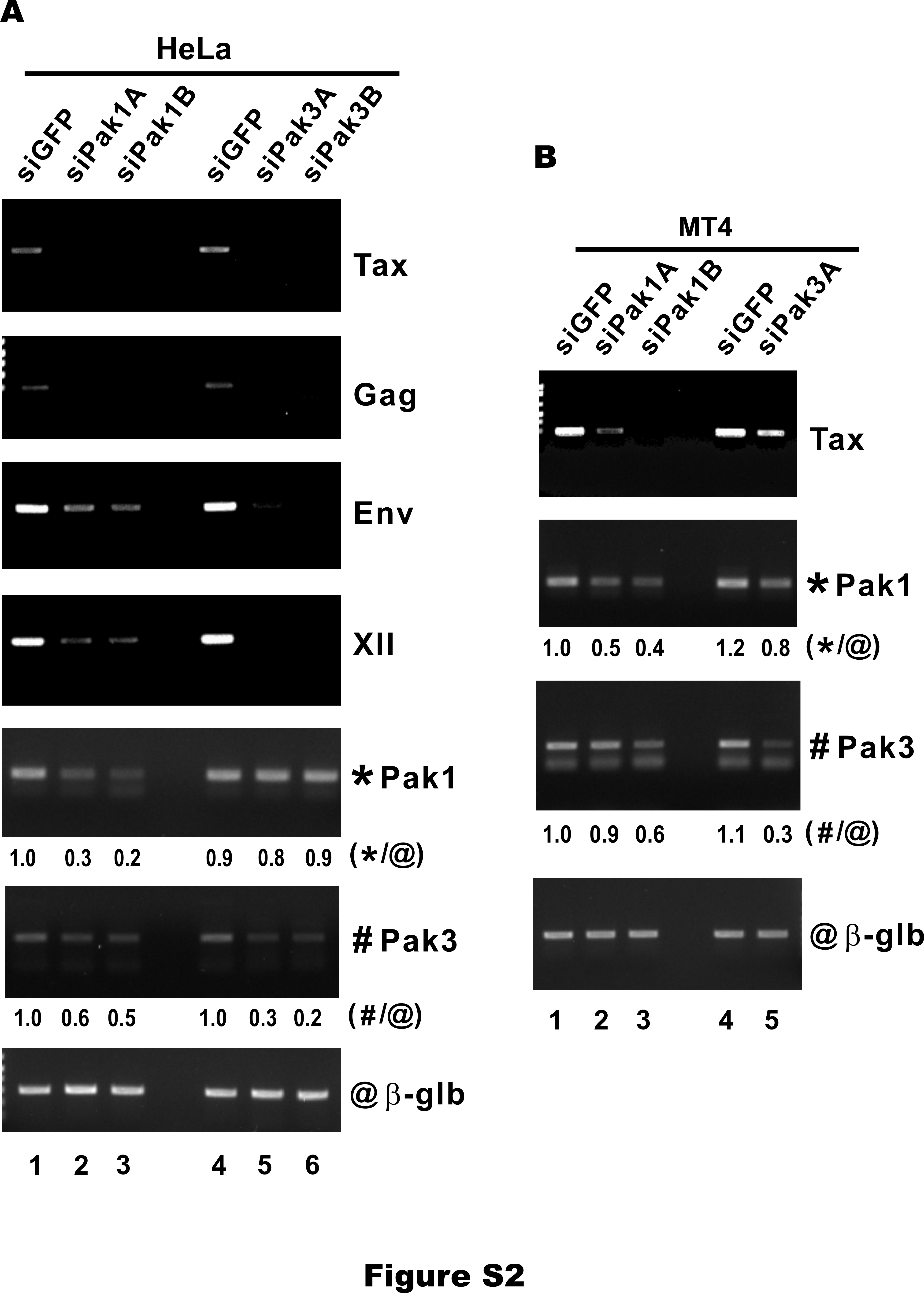

Supplement: Additional file 2: Figure S2 — Repression of HTLV-1 proviral gene transcription by depleting Pak1/3. HeLa (A) and MT4 (B) cells were co-transfected as in Figure 7. Semi-quantitative RT-PCR was performed. Indicated at the bottom of the panels are relative amounts of Pak1 or Pak3 transcript normalized to β-globin (β-glb) mRNA (*/@ and #/@) as determined by densitometric analysis of band intensity. [file 1742-4690-10-47-S2.tiff]

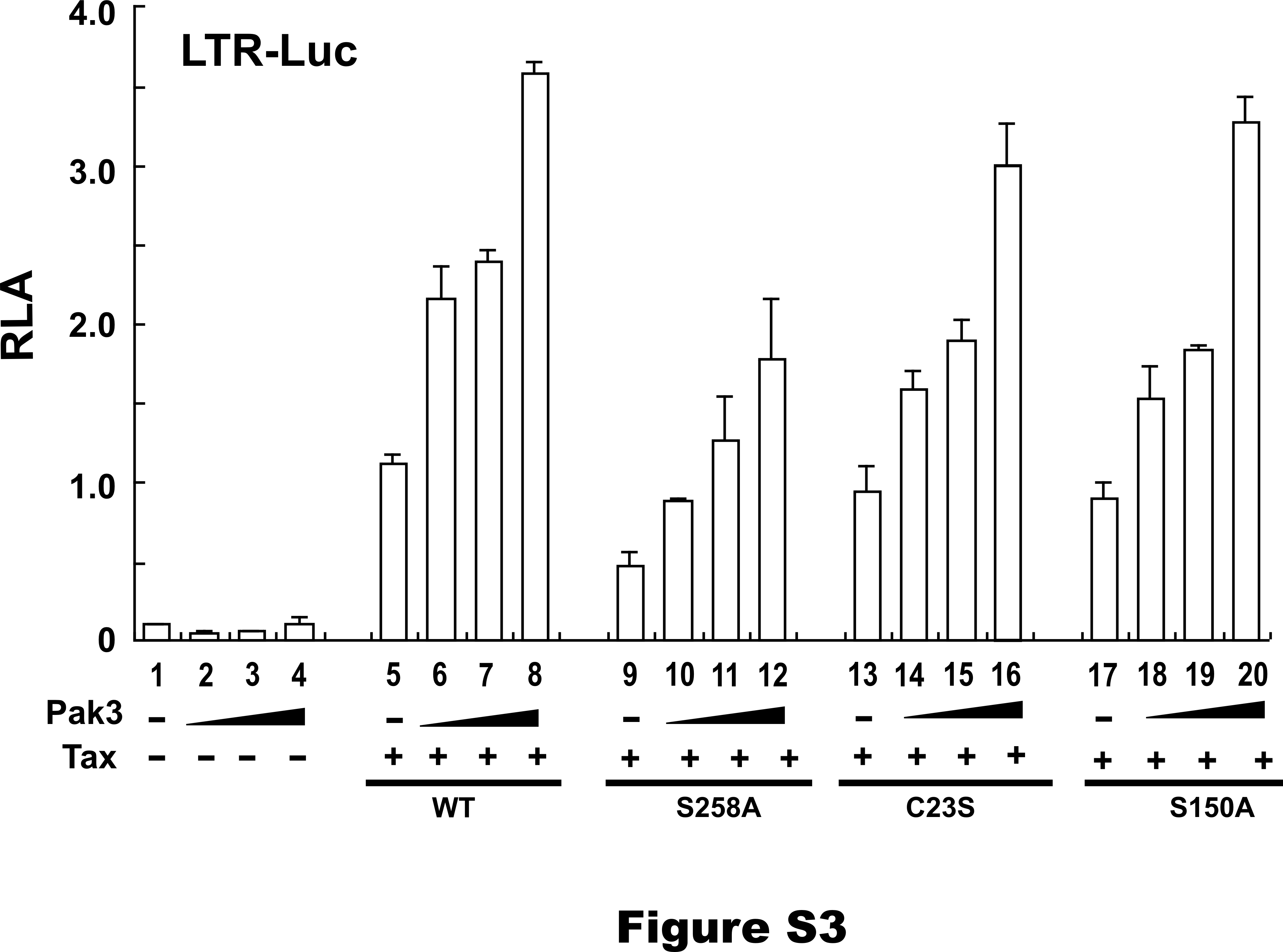

Supplement: Additional file 3: Figure S3 — Pak3 augments LTR activation by Tax mutants. HeLa cells were transfected with the indicated plasmids and analyzed as in Figure 1. [file 1742-4690-10-47-S3.tiff]

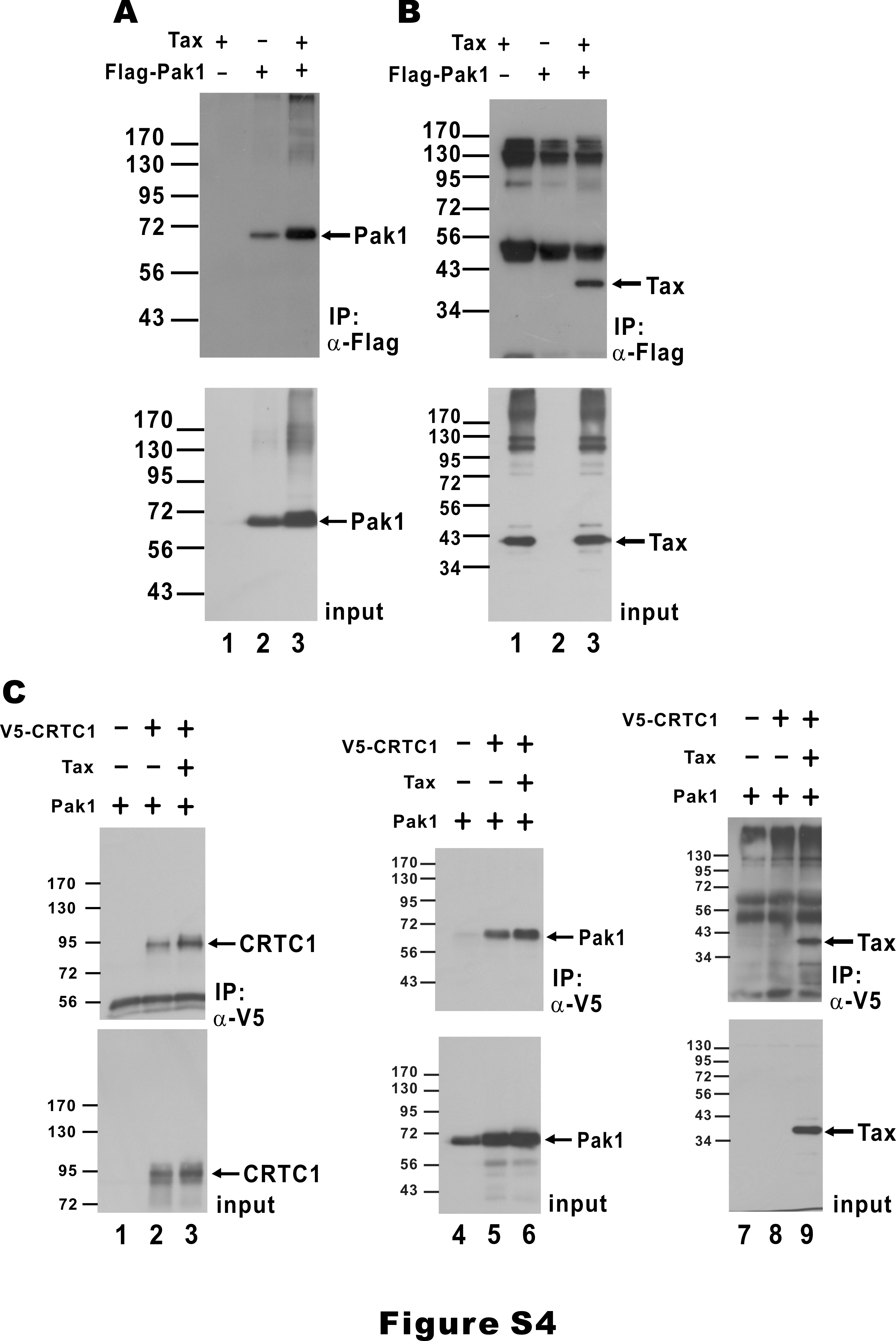

Supplement: Additional file 4: Figure S4 — Pak1 interacts with Tax and CRTC1 in HEK293T cells. Cells were co-transfected with expression vectors for Pak1, CRTC1 and Tax as indicated. Co-immunoprecipitation and Western blotting were performed as in Figure 5. [file 1742-4690-10-47-S4.tiff]
